# Addressing Food Insecurity: Lessons Learned from Co-Locating a Food Pantry with a Federally Qualified Health Center

**DOI:** 10.5334/ijic.6430

**Published:** 2022-09-30

**Authors:** Deanna Reinoso, Dawn Haut, Stephen Claffey, Kathy Hahn Keiner, Alejandra Chavez, Nicole Nace, Amy Carter

**Affiliations:** 1 Eskenazi Health Center Pecar, Indianapolis, Indiana, US; 2 Department of Pediatrics, Indiana University School of Medicine, Indianapolis, Indiana, US; 3 Crooked Creek Food Pantry, Inc., Indianapolis, Indiana, US; 4 Gleaners Food Bank of Indiana, Indianapolis, Indiana, US; 5 Corteva Agriscience LLC, Indianapolis, Indiana, US; 6 Eskenazi Health, Indianapolis, Indiana, US

**Keywords:** integrated care, food insecurity, nutrition, primary care, Federally Qualified Health Centers

## Abstract

**Introduction:**

Social determinants of health, such as food insecurity, contribute to chronic health conditions, decreased quality of life, and health disparities. Increasingly, healthcare systems seek to address social determinants of health by integrating medical and social care.

**Description:**

Eskenazi Health Center Pecar is a Federally Qualified Health Center providing comprehensive primary care to vulnerable patients in Indianapolis, IN, USA. This health center, in coalition with community partners, established and continually developed an integrated food pantry model to address food insecurity, improve nutrition education, and support patient access to healthy food.

**Discussion:**

Food insecurity and poor nutrition are common in primary care and contribute to the incidence and outcomes of chronic conditions such as obesity, hypertension, and diabetes. Long-term management of food assistance and nutrition programs requires substantial resources, partnerships, and leadership. We describe lessons learned in food pantry partnership, funding, logistics, and sustainability in a collaborative food access model integrated into healthcare. These lessons learned can be utilized by other health systems to scale up and accelerate strategies to better address food security and nutrition education. This paper articulates best practices for integrating a food pantry model within primary care with the goal of long-term sustainability and direct impact on patient health outcomes.

## Introduction

According to the World Health Organization, social determinants of health account for 30–50% of health outcomes and have a major influence on health disparities [1]. These social factors include economic stability, environment, social context, and food security among others. Feeding America projects that 42 million people (1 in 8), including 13 million children (1 in 6), in the United States may experience food insecurity in 2021 [2]. A food-insecure household is defined by the United States Department of Agriculture (USDA) as one in which “access to adequate food is limited by a lack of money and other resources” [3]. Food insecurity and poor nutrition contribute to chronic medical and mental health conditions, poor health outcomes from these conditions, health disparities, and increased healthcare costs [4,5,6,7,8,9,10,11,12,13,14,15,16,17]. The US National Academies of Science, Engineering, and Medicine recommend that healthcare organizations take steps to integrate care for social determinants of health into medical care systems. Integration of social care and medical care, including nutrition, can lead to better health outcomes for chronic conditions, and even achieve remission for chronic conditions such as hypertension and diabetes [18]. In the United States, Federally Qualified Health Centers (FQHC) seek to “deliver high quality, culturally competent, comprehensive primary care, as well as supportive services” [19]. Funded by the US Government, the program provides care to nearly 10% of the US population, including 30% of those living in poverty [20]. The Eskenazi Health FQHC comprises the largest FQHC network in the state of Indiana, delivering primary care and a broad range of other medical and social services in 14 neighborhood sites in more than 350,000 visits per year. One of these sites, Eskenazi Health Center Pecar, serves a neighborhood characterized by a large population of new immigrant families with a high prevalence of food insecurity.

Food pantries are an essential part of the food landscape for many communities and individuals who are food insecure. A food pantry can not only serve as an emergency source of nutrition but can also provide a significant portion of a participant’s dietary intake [21]. Although the food pantry model does not in itself resolve the complex root causes of food insecurity, it does provide an opportunity for improved diet quality for participants [22]. In a systematic review of food pantries in the US, An et al. suggests that in addition to emergency food provision food pantries could act as a location for additional services to be delivered that address not only diet but health status as well [28]. A commonly accepted model for addressing food insecurity in medical care settings starts with patient screening followed by referral to appropriate intramural nutrition education resources and/or referral to external community-based organizations offering food or related services [23,24,25]. This model is often inadequate. As recently described in a study of urban food pantries, patients often do not connect with these community pantries due to medically inappropriate or unacceptable food items, inaccessibility due to hours of operation or location, or inadequate volume of food [26]. Community members face barriers such as transportation, time constraints, and the social stigma associated with food insecurity and primary care providers report system level barriers within the medical practice [27]. This Integrated Care Case Study describes the six-year experience of developing and implementing a large-scale food pantry co-located at the Pecar FQHC site in partnership with the local neighborhood and multiple community-based organizations. While there have been other successful integrated efforts to address food insecurity, health systems often underappreciate the importance of sustainable and comprehensive partnerships to meet the complex and changing food access needs for all individuals in a community [14,28,29].

## Description of the care practice

A comprehensive approach to addressing food insecurity addresses not only hunger, but also the full spectrum of poor nutrition leading to common chronic conditions. Paradoxically, food insecurity may lead to obesity by pushing families toward low-cost, high-calorie, low-nutritional value foods [30,31]. Thus, food pantries seek to provide not simply calories, but nutritious foods. By addressing hunger with a multidisciplinary and community partnership approach, we also hope to move the patient or client toward improved nutrition and the capacity to maintain a healthy diet at home. The “Crooked Creek Food Pantry” was established in 2015 at Eskenazi Health Center Pecar within the same building where all other FQHC health services are provided. The co-location of CCFP within the health center provides patients with quick, convenient, and immediate access to healthy food options in response to screening positive for food insecurity. The proximity of CCFP also provides multiple opportunities for clinic staff to link patients with other specific health concerns or health risks related to nutrition to the appropriate food resources. From the outset, Crooked Creek Food Pantry (CCFP) partnered with Gleaners Food Bank of Indiana (Gleaners). As the largest hunger-relief organization in Indiana, Gleaners distributes food to a network of over 300 agencies throughout its 21-county service area, including food pantries, emergency soup kitchens, schools, and community partners. With its 297,000 square foot facility, fleet of trucks, and material handling equipment, Gleaners is afforded tremendous capacity to distribute large volumes of food efficiently and safely for the hungry. This partnership with Gleaners and multiple other key community partners immediately provided synergy in terms of access, scalability, and sustainability by utilizing the strengths of all the organizations involved.

By the end of its first year of operation in 2015, CCFP had served 5,284 household visits (21,256 individuals based on client reported household size) and in 2019 that number grew to a total of 18,443 household visits served (85,650 individuals). With the SARS-CoV2 global pandemic, CCFP increased capacity to serve 24,186 household visits (95,404 individuals) in 2020, 25,877 household visits (102,321 individuals) in 2021 and is now projecting the number of household visits served for 2022 to far exceed these numbers. Additionally, when the clinical care teams implemented the integrated workflow process for food insecurity screening in 2019, the volume of Eskenazi Health Center Pecar specific patient referrals utilizing CCFP started to rapidly increase (Table 1).

**Table 1:** Data for Crooked Creek Pantry by year.


YEAR	VISITS (HOUSEHOLDS)	INDIVIDUALS SERVED	CLINIC REFERRALS SERVED

2015	5,284	21,256	1,298

2016	10,376	42,657	2,744

2017	12,184	71,935	2,847

2018	11,686	44,467	2,953

2019	18,443	85,650	4,780

2020	24,186	95,404	6,672

2021	25,877	102,321	9,659


### Workflow and Electronic Health Record Infrastructure

A key sustainability feature of this model is a goal to embed the care processes for food access and nutrition within the current health system workflow. Annual screening was implemented in 2019 for patients receiving care at Eskenazi Health Center Pecar utilizing the two validated Hunger Vital Sign^TM^ questions. Prior research demonstrates these questions to be sensitive and specific for identifying households at risk for food insecurity [32,33,34]. The responses to these screening questions are recorded in the Epic® electronic health record (EHR). The EHR interface highlights food security status in a Social Determinant of Health (SDOH) display visible to the provider. Since implementation of food insecurity screening infrastructure in 2019 at Eskenazi Health Center Pecar FQHC, more than one-third of patients screened positive (Table 2).

**Table 2:** Eskenazi Health Center Pecar Screening and Nutrition Services by Year.


YEAR	PATIENT VISITS SCREENED FOR FI	PATIENT VISITS SCREENED POSITIVE FOR FI	PERCENTAGE POSITIVE FI SCREENS	INDIVIDUAL PATIENT DIETICIAN VISITS	FOOD IS MEDICINE/LIFESTYLE GROUP PARTICIPANTS	VEGGIE BOX PARTICIPATION

**2018**	*	*	*	1447	*	*

**2019**	3,397	1,025	30.20%	1864	41	25

**2020**	4,689	1,734	37.00%	2123	34	21

2021	5,826	2,157	37.00%	3011	37	37


*Food Insecurity (FI) Screening, Food is Medicine/Lifestyle Group Visits and Veggie Box Program all implemented 2019.

**Figure 1:**
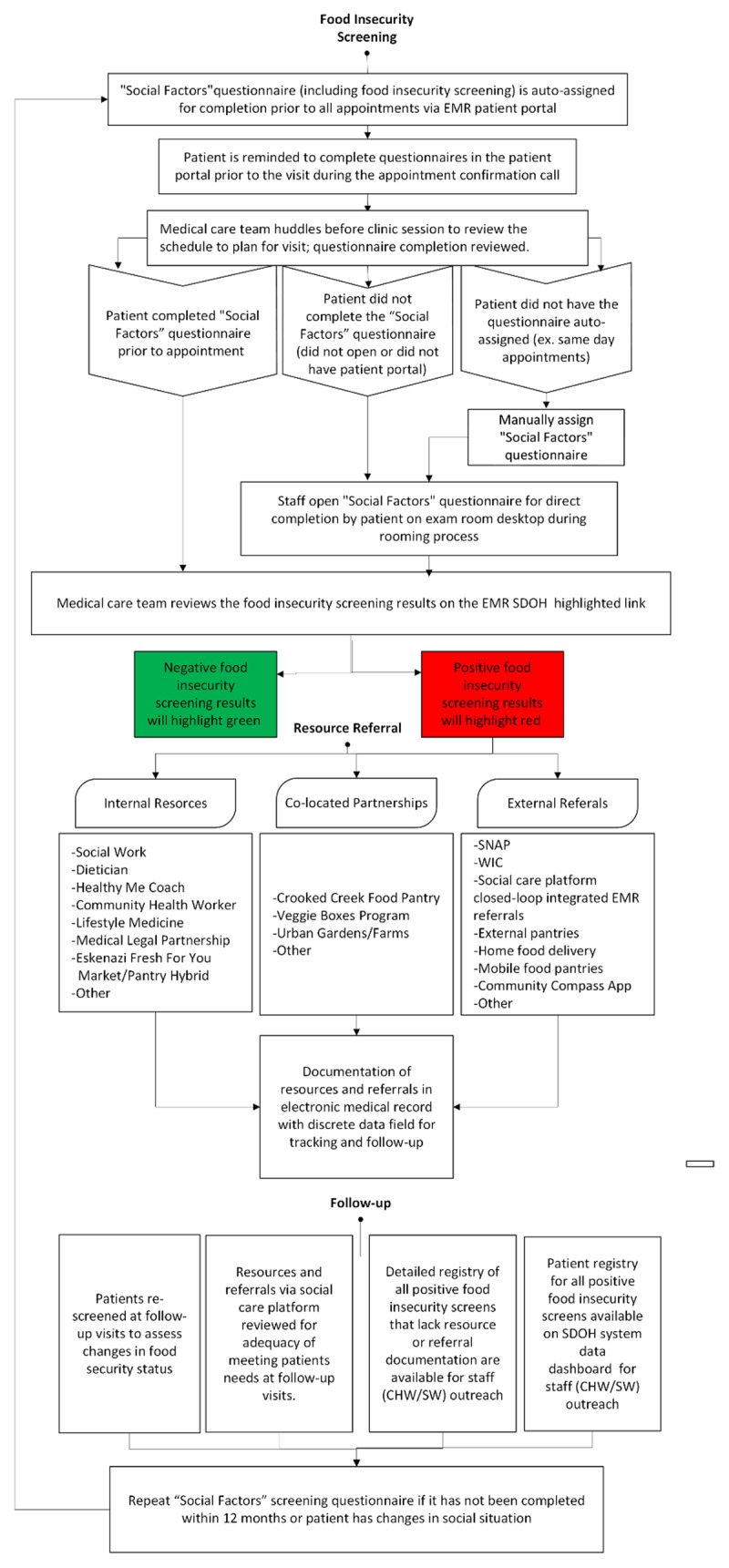
Food Insecurity Screening and Referral Workflow.

Additional EHR-based tools support providers’ electronic referral to other social care and nutrition services both within and outside Eskenazi Health. These may include internal referrals to social workers, dietitians, health coaches, application support for the US Supplemental Nutrition Assistance Program (SNAP), or financial counseling. Referrals may also include food pantry resources, free prepared meals, Supplemental Nutrition Program for Women, Infants and Children (WIC), SNAP, and other community food services that can be accessed on the Indy Hunger Network’s “Community Compass^TM^” smart phone-based food access guide. The EHR captures these referrals and provides a visual reminder if a positive screen does not have an associated documented resource assistance referral. These EHR support tools help to assure follow-up to determine if the patient was able to access and utilize the recommended food resources. One final aspect of the EHR infrastructure includes support for a team-based approach to health care. As noted, our goal is to embed the workflow of care for social determinants within the same teams and workflow for other health conditions. This requires a communication platform that also supports a primary care culture that assigns responsibility, accountability, and resources to specific team members. Hospital inpatient, specialty care, emergency department, and mental health care teams also utilize the same EHR screening platform, so documentation is visible throughout the health system. A clear process and role for each clinical team member is necessary to effectively administer screening questions, review results, refer patients who need resources, and follow-up whether food access needs were met. For example, with about one-third of all patients screening positive for food insecurity, clinic-based social workers cannot meet the patients’ needs without a team approach. Figure 1 displays the flow of patients through the screening and referral process.

**Figure 2:**
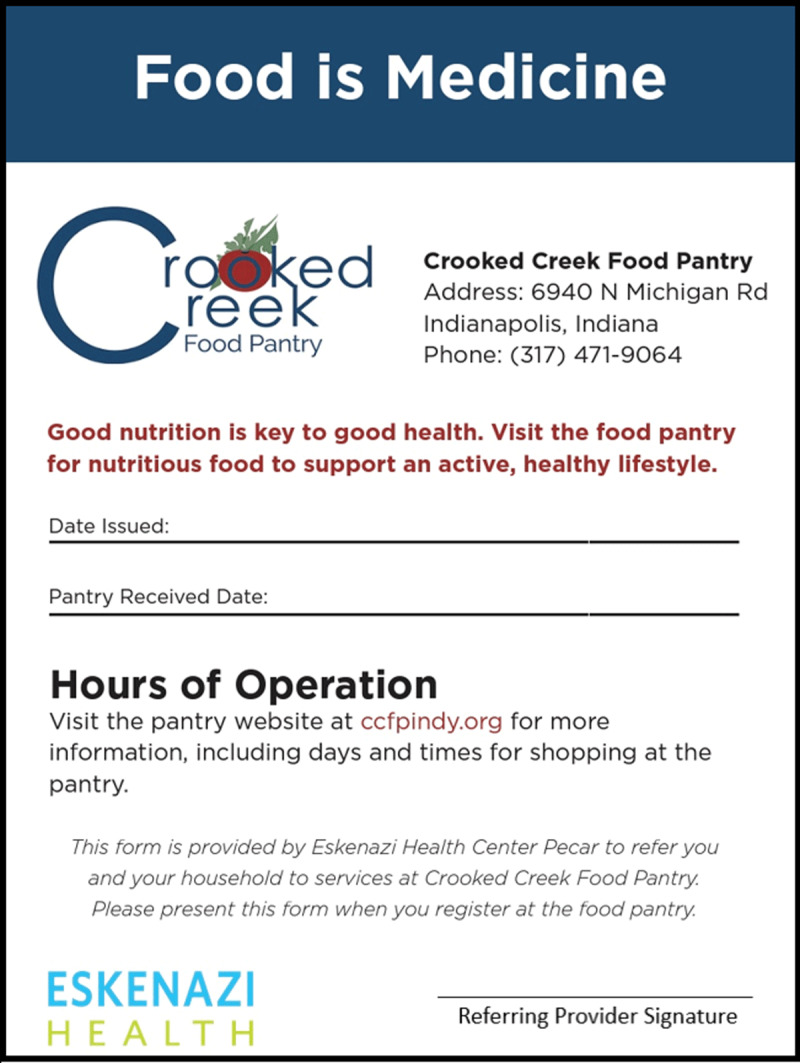
“Food is Medicine” paper referral.

Food insecure patients receive a “Food is Medicine” paper referral to present for registration at the CCFP (see Figure 2). We highlight that this “food prescription”, as well as the co-located pantry with embedded nutrition education resources, strives to decrease stigma associated with food insecurity and overcome multiple other access barriers. This referral process focuses on access to nutritious food as part of the medical care plan rather than focusing on the reported food insecurity. Additionally, nutrition education with a multidisciplinary “Food is Medicine” group visit model at CCFP provides an inclusive pantry environment focused on improving nutritional health and well-being. Since the inception of the “Food is Medicine” group visit model, there have been 12 separate groups facilitated, with a total of 112 clients participating in the eight-week series. The “Food is Medicine” program is offered for both English and Spanish language groups with individuals participating in the groups self-identifying as 55% Hispanic and 45% non-Hispanic. Responses on pre-program and post-program surveys indicate increased consumption of fruits and vegetables following program completion. At graduation, 73% of participants reported consuming 3 or more servings of fruits daily compared with 56% at enrollment. Daily vegetable intake of 4 or more servings per day increased to 58% of participants at program completion from 45% at enrollment. Participants also build shopping skills and cooking confidence through interactive learning and cooking demonstrations. At graduation, 80% agree that they have the shopping skills needed to buy healthy food that fits into their household budget and 82% express confidence in cooking skills showing an increase near 15% in both areas. During the SARS-CoV2 pandemic the programming and group visits shifted to virtual classes with subsequent CCFP food pick up coordinated on site. Despite the sudden change to virtual programming for the “Food is Medicine” groups, they continued to be a successful and well attended model of care with partnership between the CCFP and Eskenazi Health Center Pecar (Table 2).

Patients can choose to enroll in other nutrition education programs that are an essential part of a comprehensive approach to food insecurity. CCFP includes a mobile education kitchen where groups of patients and their families can learn to cook with a health center registered dietitian and taste nutritious recipes or learn how to follow medically tailored diets. Health center dietitians also engage with patients with education about food preparation, cooking, nutrition and food label assessment, grocery shopping, and disease-specific nutrition guidance. This extensive nutrition education programming is made possible by the co-located food pantry in a health care center. Monthly food demonstrations by the registered dietitians at CCFP began in 2016 and continued until the “Food Is Medicine” group visits started in 2019. During this period, there were 29 food demonstrations for general shoppers, with an estimated 2175 clients attending. An additional outreach during senior shopping hours included 22 demonstrations with 770 attending. While nutrition programming continued to develop at CCFP, it also continued to drive the increase in individual dietitian patient visits at Eskenazi Health Center Pecar. The Eskenazi Health Center Pecar dietitians (1.5 full time employment) saw volumes more than double over the four-year time period (Table 2). Registered dietitians are also able to work along with the patients’ primary care team to best utilize pantry resources to meet any special dietary needs and further develop nutritional goals based on their medical diagnosis.

Households register to shop at the co-located CCFP for nutritious food items twice a month as needed. Prior to the pandemic, CCFP clients utilized a “client choice” food pantry model with availability of a variety of fresh produce, frozen food items, eggs, milk, and other staple items partially based on family size. During the SARS-CoV2 pandemic CCFP distributed the same items to households in a drive -through system at the same health center site, but without the individual personal shopping choice experience. Households received about 80 pounds (average) of food with the initial “client choice” model and now with the drive through pantry model each household receives an average is about 140 pounds of food. This approximate 50% increase in food distributed in the drive -through model has allowed households to get more food without increasing number of pantry visits. Other resources from community-based organizations, community gardening efforts, and other food resources and social services are also provided to families with additional needs.

### Engaging the Community

The Crooked Creek Food Pantry is a separate 501c3 nonprofit corporation and is led by a volunteer Board of Directors with representation from the founding member organizations of Corteva Agriscience (formerly Dow Agroscience), Eskenazi Health, and St. Luke’s United Methodist Church, as well as other key community partners. It receives donations and purchased food from multiple food banks and local business partners in addition to Gleaners. CCFP also purchases at retail about 25% of the food it distributes. Individual volunteers represent one of the most important resources required for managing and sustaining a food pantry in a health facility. Currently CCFP is managed by one full-time volunteer Executive Director, two full-time paid managers, one part-time interpreter, one part-time truck driver, and about 120 volunteers per week. Total CCFP staff and volunteer work hours exceed 500 hours per week. Volunteer capacity, training, scheduling, and organization are significant tasks that are essential for a large food pantry to function at full capacity. For example, to support obtaining the quantities of food required for ample distributions of food to 2,850 households a month, CCFP facilitates up to 44 separate food pickups weekly. In addition to the work needed to organize the volunteers, we summarize the volunteer workforce needed to maintain pantry function and capacity as follows: 1) Unloading, boxing, and bagging volunteers total about 50 per week, 2) Food distribution volunteers total about 40 per week, 3) Registration and sign-in volunteers total about 10 per week, and 4) Cardboard and trash management and general cleanup requires 10 volunteers per week. Our partner organizations facilitate and encourage volunteer efforts, as well as financial donations, donation of other food resources, and infrastructure resources.

## Discussion

We highlight four lessons learned to guide others seeking to integrate medical care and social care to address food insecurity: partnership, funding, logistics, and sustainability.

### Partnership

Well-intentioned efforts without partnership with community-based organizations and the community served can exacerbate and amplify the harmful impacts of food insecurity. As health systems become more aware of the significant impact food insecurity has on individual health outcomes and health disparities, they often seek quick but unsustainable solutions. A common example of such a solution includes food closets supported through food donations from medical staff and their families. These food sources typically cannot provide a balanced source of nutrition or even enough low nutritional value calories to sustain food insecure families for weeks at a time. A small capacity food pantry with shelf-stable canned and dry food products that are high in sodium and carbohydrate content are often contrary to the dietary recommendations for patients with chronic health conditions. Another example of an inadequate response to individuals who are food insecure is the simple provision of names and contact information for food pantry organizations in the local community. As noted earlier, patients face multiple additional access hurdles without facilitated referrals to community-based food related partners. Finally, these smaller efforts often duplicate larger scale, more efficient (albeit potentially less accessible) food pantry operations. When viewed from the perspective of the full range of social determinants of health, health systems must develop and manage partnerships with organizations whose capacity, efficiency, and expertise in any particular area (food, housing, transportation, financial stability, education, etc.) exceeds that of the health system.

### Funding

Partnerships also provide a foundational level of funding support both in terms of financial assistance from larger organizations and donations from individual donors. Notably, significant leadership effort and strategic planning is needed for sustainable funding of a large-scale food pantry. The community need for food access in the FQHC setting is high and funding a large food pantry takes a commitment from the health system and community partners. Initial funding for CCFP was obtained well in advance of the pantry opening and continues to evolve to meet the ongoing funding demands. Over the past six years funding sources include extramural grants, corporations, faith-based organizations, and individual philanthropy. Direct food donations are a large aspect of sustainability for CCFP. In 2015 CCFP had total expenses of $60,000 (US). In the first full year of operation in 2016 the donation amount breakdowns were as follows: Direct Food Donations $301,000, Individual Food Donations $22,000, Faith-Based Donations $93,000, and Corporate Donations $95,000, for a total of $511,000. Currently the annual budget exceeds $360,000 (US) which does not include the significant estimated retail value of donated food that now exceeds $2.5 million (US) annually. In any given year, annual expenses may exceed these figures as the number of clients served increases and as the program is awarded funding for capital expenditures, vehicles and/or other equipment.

Additional funding sustainability was added in September 2019 when CCFP began to participate in The Emergency Food Assistance Program (TEFAP). TEFAP is a federal program that helps supplement the diets of low-income Americans by providing them with emergency food assistance at no cost. The United States Department of Agriculture (USDA) provides food and administrative funding to states to operate TEFAP [35]. With this new affiliation CCFP started receiving between $20,000-$30,000 retail value worth of food monthly from the USDA. This additional support allows for increased volume of food distributed to each client, while decreasing food procurement costs. Unfortunately, it is unknown how long this volume of benefit will continue, and one of the potential sustainability challenges could be adjusting to a potential reduction of TEFAP donations. Additionally, in 2020 due to COVID-19, the USDA implemented the Coronavirus Food Assistance Program (CFAP) to provide financial assistance to farmers, ranchers, and producers impacted by market disruptions. The CFAP funding allowed for additional distribution of food produced by U.S. farmers to local food insecure individuals via CCFP.

### Logistics

The space needed for food storage and pantry shopping was also initially underestimated. In July 2018 CCFP doubled the original square footage for client shopping and inventory storage to approximately 2,500 square feet. Additional issues that required a larger space included increased space needed for clients waiting to shop in the food pantry, larger entry space for food pallet delivery, and additional pallet and shopping cart storage space. CCFP generated large volumes of cardboard and food packaging materials, as typically occurs with food pantries, which necessitated additional recycling and dumpster capacity.

To highlight some of these logistical challenges, we specifically note the demand for refrigeration and freezer capacity. CCFP began operations in 2015 with a total of 230 cubic feet of refrigeration and freezer capacity. This cooling and freezer capacity proved inadequate. Over the following two years, CCFP expanded refrigeration to 400 cubic feet. This refrigeration and freezer capacity remained inadequate for the number of individuals served. The commitment to provide healthy food options that need refrigerator or freezer storage led the food pantry to eventually increase its cooling capacity over its first six years of operation from 230 cubic feet in 2015 to approximately 1,605 cubic feet.

Since CCFP opened, there have been several adjustments in the food distribution approach to take into consideration the household size. Initially in 2015, virtually every category of food had a selection level based on the household size allowing more food for larger families. As the pantry volumes grew, it became apparent that it was difficult for volunteers to manage the pantry flow to include the variability in number of items to be allowed for each category based on household size. A shift was made to reduce the number of items that were household size sensitive. Another change to tailored distribution of food volumes for varying household sizes was necessitated by the SARS CoV2 pandemic. Currently all households receive larger but equal food distributions independent of household size. There will likely be further process adjustments necessitated as the pantry food distribution model continues to adapt to changes and constraints while still ensuring the community need for food pantry services is met. We highlight the value of partnerships with large organizations with substantial logistic expertise and workforce capabilities to underscore the importance of these partnerships in responding to change.

### Sustainability

As already noted, labor, funding, and logistic support represent major challenges to sustainability. However, within each of these areas, sustainability also requires innovative approaches to old problems and novel approaches to unanticipated or new problems. The COVID-19 pandemic disrupted operations; it also caused a large surge of food insecure individuals in the community. In order to further sustain sufficient food supply to meet the increased demand, CCFP utilized additional federal resources with The US Emergency Food Assistance Program TEFAP and the Coronavirus Food Assistance Program (CFAP) as described above in funding section. There was also an effort to greatly increase the network of retail and food bank donation partners as another approach to obtain sufficient food supply to meet the increasing demand for food pantry services.

The sustainability of food insecurity screening and the social determinants of health workflow implementation has largely relied on significant health system investment in the built infrastructure, food resource partnership and staff development. It has been essential to streamline and decrease the human resource burden of screening and resource referral by supporting the process within existing workflows in the electronic medical record. A team-based care workflow approach to food insecurity screening and referral helps to reduce the burden on any individual health provider. Development of a dashboard for social determinants of health data collection allows for the incorporation of quality metrics such as food insecurity screening rates into individual performance goals as another approach to sustaining the workflow process and high screening rates. Investment in the social determinants of health system leadership structure and the integration of additional community outreach positions, such as community health workers and community weavers, has accelerated and sustained efforts to address community social health needs.

Additionally, the sustainability of this food pantry model integrated into Eskenazi Health Center Pecar is enhanced by being part of a larger health system and a public entity of Health & Hospital Corporation (HHC) of Marion County. Each of the fourteen Eskenazi FQHC sites are a part of this larger FQHC co-applicant enterprise that provides access to additional resources that a typical independent FQHC may not be able to provide. For example, the larger Eskenazi Health and HHC system allowed access to additional food pantry staffing and other support needed during the SARS-CoV2 global pandemic. The “in kind” donation of health center space, utilities, and additional infrastructure support are all benefits of being a part of a larger health system. Another essential ingredient to the success and sustainability of this food access model is the understanding and unwavering support of the physician-led Eskenazi Health senior leadership and mission for a comprehensive approach to food as medicine, lifestyle medicine, and all the other social determinants of health.

## Conclusion

Chronic health conditions and long-term health outcomes are significantly impacted by nutrition, so it is imperative that every health system has a universal food insecurity screening infrastructure and process as well as a comprehensive food resource and nutrition education strategy. Despite the essential role that food pantry models serve in providing emergency nutrition resources, it is understood that as an isolated intervention they do not resolve many of the complexities of food insecurity and the associated risk of chronic disease. Addressing food access disparities is essential to improving the health and well-being of every individual in the community, yet most health systems have minimal experience or expertise in the implementation of a sustainable food access model integrated into primary health care. Health systems historically have grossly underestimated the large capacity and extensive partnerships necessary to sustain an effective food access strategy. High quality food resources and nutrition education that are comprehensive, culturally sensitive, patient-centered, accessible, and facilitated with community partnership in the health care setting are essential but exceedingly difficult for health systems to implement and sustain. Well-intentioned efforts by health systems to provide small pantry capacity, without consistent nutritious food options, may further perpetuate and worsen the poor health outcomes related to the inadequate nutrition of those who are food insecure. Eskenazi Health Center Pecar and Crooked Creek Food Pantry are successful examples of a healthcare and pantry partnership seeking to provide an initial solution for this key social determinant of health. It demonstrates that the linkage of a health center with a food pantry is a powerful and innovative way to begin to effect change in the health and well-being of a community. Health systems can utilize the lessons learned from the implementation of this large-scale, co-located food pantry partnership to avoid common pitfalls and unsustainable food access strategies.

Further innovation and study of best practices in addressing food access and nutrition education are needed as health systems partner with communities to address health disparities and barriers related to food insecurity. Partnerships outside of the traditional healthcare establishment are essential with the recognition that many community-based partners have the extensive experience and expertise that healthcare systems typically lack in the management of food pantry and food access resources. Community food pantry partners value the health care systems’ ability to screen for food insecure individuals, understand the complexities of individuals health and social situation, provide expertise in nutrition-related disease management, and nutrition education. Effective partnership and integrated care will better address the impact of food insecurity on the health of individuals and communities for improved health outcomes and decreased nutritional-related health disparities.

## References

[1] World Health Organization. Social determinants of health; 2021. Available from: https://www.who.int/health-topics/social-determinants-of-health.

[2] Feeding America. Hunger in America; 2021. Available from: https://www.feedingamerica.org/hunger-in-america.

[3] Coleman-Jensen A, Rabbitt MP, Gregory CA, Singh A. Household Food Security in the United States in 2018. In: U.S. Department of Agriculture, ERS, (ed.); 2019.

[4] Shankar P, Chung R, Frank DA. Association of Food Insecurity with Children’s Behavioral, Emotional, and Academic Outcomes: A Systematic Review. Journal of Developmental and Behavioral Pediatrics. 2017; 38(2): 135–50. DOI: 10.1097/DBP.0000000000000383 28134627

[5] Carmichael SL, Yang W, Herring A, Abrams B, Shaw GM. Maternal food insecurity is associated with increased risk of certain birth defects. The Journal of Nutrition. 2007; 137(9): 2087–92. DOI: 10.1093/jn/137.9.2087 17709447 PMC2063452

[6] Nagata JM, Palar K, Gooding HC, Garber AK, Whittle HJ, Bibbins-Domingo K, et al. Food Insecurity Is Associated With Poorer Mental Health and Sleep Outcomes in Young Adults. The Journal of Adolescent Health. 2019; 65(6): 805–11. DOI: 10.1016/j.jadohealth.2019.08.010 31587956 PMC6874757

[7] Koyanagi A, Stubbs B, Oh H, Veronese N, Smith L, Haro JM, et al. Food insecurity (hunger) and suicide attempts among 179,771 adolescents attending school from 9 high-income, 31 middle-income, and 4 low-income countries: A cross-sectional study. Journal of Affective Disorders. 2019; 248: 91–8. DOI: 10.1016/j.jad.2019.01.033 30716616

[8] Mangini LD, Hayward MD, Dong YQ, Forman MR. Household Food Insecurity is Associated with Childhood Asthma. The Journal of Nutrition. 2015; 145(12): 2756–64. DOI: 10.3945/jn.115.215939 26491120

[9] Koskinen J, Magnussen CG, Sinaiko A, Woo J, Urbina E, Jacobs DR Jr., et al. Childhood Age and Associations Between Childhood Metabolic Syndrome and Adult Risk for Metabolic Syndrome, Type 2 Diabetes Mellitus and Carotid Intima Media Thickness: The International Childhood Cardiovascular Cohort Consortium. Journal of the American Heart Association. 2017; 6(8). DOI: 10.1161/JAHA.117.005632 PMC5586423 28862940

[10] Zhu Y, Mangini LD, Hayward MD, Forman MR. Food insecurity and the extremes of childhood weight: defining windows of vulnerability. International Journal of Epidemiology. 2020; 49(2): 519–27. DOI: 10.1093/ije/dyz233 31750907 PMC7266558

[11] Banerjee T, Crews DC, Wesson DE, Dharmarajan S, Saran R, Rios Burrows N, et al. Food Insecurity, CKD, and Subsequent ESRD in US Adults. American Journal of Kidney Diseases. 2017; 70(1): 38–47. DOI: 10.1053/j.ajkd.2016.10.035 28215947 PMC5765854

[12] Nagata JM, Palar K, Gooding HC, Garber AK, Bibbins-Domingo K, Weiser SD. Food Insecurity and Chronic Disease in US Young Adults: Findings from the National Longitudinal Study of Adolescent to Adult Health. Journal of General Internal Medicine. 2019; 34(12): 2756–62. DOI: 10.1007/s11606-019-05317-8 31576509 PMC6854148

[13] Gundersen C, Ziliak JP. Food Insecurity And Health Outcomes. Health Affairs (Millwood). 2015; 34(11): 1830–9. DOI: 10.1377/hlthaff.2015.0645 26526240

[14] Eicher-Miller HA. A review of the food security, diet and health outcomes of food pantry clients and the potential for their improvement through food pantry interventions in the United States. Physiology & Behavior. 2020; 220: 112871. DOI: 10.1016/j.physbeh.2020.112871 32179054

[15] Walker RJ, Garacci E, Ozieh M, Egede LE. Food insecurity and glycemic control in individuals with diagnosed and undiagnosed diabetes in the United States. Primary Care Diabetes; 2021. DOI: 10.1016/j.pcd.2021.05.003 PMC8458221 34006474

[16] *Integrating social care into the delivery of health care: moving upstream to improve the nation’s health*. Washington, DC: The National Academies Press; 2019. 31940159

[17] Liu Y, Eicher-Miller HA. Food Insecurity and Cardiovascular Disease Risk. Curr Atheroscler Rep. 2021; 23(6): 24. DOI: 10.1007/s11883-021-00923-6 33772668 PMC8000689

[18] Lean ME, Leslie WS, Barnes AC, Brosnahan N, Thom G, McCombie L, et al. Primary care-led weight management for remission of type 2 diabetes (DiRECT): an open-label, cluster-randomised trial. Lancet. 2018; 391(10120): 541–51. DOI: 10.1016/S0140-6736(17)33102-1 29221645

[19] Health Resources & Services Administration. What is a Health Center? 2021. Available from: https://bphc.hrsa.gov/about/what-is-a-health-center/index.html.

[20] Health Resources & Services Administration. HRSA Health Center Program. Available from: https://bphc.hrsa.gov/about/healthcenterprogram.

[21] Liu Y, Desmond NE, Wright BN, Bailey RL, Dong T, Craig BA, et al. Nutritional contributions of food pantries and other sources to the diets of rural, Midwestern food pantry users in the USA. Br J Nutr. 2021; 125(8): 891–901. DOI: 10.1017/S0007114520003372 32873361 PMC11800361

[22] Liu Y, Zhang Y, Remley DT, Eicher-Miller HA. Frequency of Food Pantry Use Is Associated with Diet Quality among Indiana Food Pantry Clients. J Acad Nutr Diet. 2019; 119(10): 1703–12. DOI: 10.1016/j.jand.2019.02.015 31040071

[23] Ruiz Escobar E, Pathak S, Blanchard CM. Screening and Referral Care Delivery Services and Unmet Health-Related Social Needs: A Systematic Review. Prev Chronic Dis. 2021; 18: E78. DOI: 10.5888/pcd18.200569 34387188 PMC8388203

[24] Smith S, Malinak D, Chang J, Perez M, Perez S, Settlecowski E, et al. Implementation of a food insecurity screening and referral program in student-run free clinics in San Diego, California. Prev Med Rep. 2017; 5: 134–9. DOI: 10.1016/j.pmedr.2016.12.007 27990340 PMC5157787

[25] Stenmark SH, Steiner JF, Marpadga S, Debor M, Underhill K, Seligman H. Lessons Learned from Implementation of the Food Insecurity Screening and Referral Program at Kaiser Permanente Colorado. Perm J. 2018; 22: 18–093. DOI: 10.7812/TPP/18-093 PMC6175601 30296400

[26] Ginsburg ZA, Bryan AD, Rubinstein EB, Frankel HJ, Maroko AR, Schechter CB, et al. Unreliable and Difficult-to-Access Food for Those in Need: A Qualitative and Quantitative Study of Urban Food Pantries. Journal of Community Health. 2019; 44(1): 16–31. DOI: 10.1007/s10900-018-0549-2 30019196 PMC6330151

[27] Herrera CN, Brochier A, Pellicer M, Garg A, Drainoni ML. Implementing Social Determinants of Health Screening at Community Health Centers: Clinician and Staff Perspectives. J Prim Care Community Health. 2019; 10: 2150132719887260. DOI: 10.1177/2150132719887260 31702425 PMC6843733

[28] An R, Wang J, Liu J, Shen J, Loehmer E, McCaffrey J. A systematic review of food pantry-based interventions in the USA. Public Health Nutrition. 2019; 22(9): 1704–16. DOI: 10.1017/S1368980019000144 30834852 PMC10260889

[29] Gany FM, Pan S, Ramirez J, Paolantonio L. Development of a Medically Tailored Hospital-based Food Pantry System. Journal of Health Care for the Poor and Underserved. 2020; 31(2): 595–602. DOI: 10.1353/hpu.2020.0047 33410795 PMC8073793

[30] Darmon N, Drewnowski A. Contribution of food prices and diet cost to socioeconomic disparities in diet quality and health: a systematic review and analysis. Nutr Rev. 2015; 73(10): 643–60. DOI: 10.1093/nutrit/nuv027 26307238 PMC4586446

[31] Drewnowski A. Nutrient density: addressing the challenge of obesity. Br J Nutr. 2018; 120(s1):S8-S14. DOI: 10.1017/S0007114517002240 29081311

[32] Hager ER, Quigg AM, Black MM, Coleman SM, Heeren T, Rose-Jacobs R, et al. Development and validity of a 2-item screen to identify families at risk for food insecurity. Pediatrics. 2010; 126(1): e26–32. DOI: 10.1542/peds.2009-3146 20595453

[33] Baer TE, Scherer EA, Fleegler EW, Hassan A. Food Insecurity and the Burden of Health-Related Social Problems in an Urban Youth Population. J Adolesc Health. 2015; 57(6): 601–7. DOI: 10.1016/j.jadohealth.2015.08.013 26592328

[34] Gundersen C, Engelhard EE, Crumbaugh AS, Seligman HK. Brief assessment of food insecurity accurately identifies high-risk US adults. Public Health Nutr. 2017; 20(8): 1367–71. DOI: 10.1017/S1368980017000180 28215190 PMC10261547

[35] USDA Food and Nutrition Service. The Emergency Food Assistance Program; 2021. Available from: https://www.fns.usda.gov/tefap/emergency-food-assistance-program.

